# *Rickettsia conorii* Transcriptional Response within Inoculation Eschar

**DOI:** 10.1371/journal.pone.0003681

**Published:** 2008-11-10

**Authors:** Patricia Renesto, Clarisse Rovery, Jacques Schrenzel, Quentin Leroy, Antoine Huyghe, Wenjun Li, Hubert Lepidi, Patrice François, Didier Raoult

**Affiliations:** 1 Unité des Rickettsies, IRD-CNRS UMR 6236, Faculté de Médecine, Marseille, France; 2 Service of Infectious Diseases/Genomic Research Laboratory, University of Geneva Hospitals, Geneva, Switzerland; University of California Los Angeles, United States of America

## Abstract

**Background:**

*Rickettsia conorii*, the causative agent of the Mediterranean spotted fever, is transmitted to humans by the bite of infected ticks *Rhipicephalus sanguineus*. The skin thus constitutes an important barrier for the entry and propagation of *R. conorii*. Given this, analysis of the survival strategies used by the bacterium within infected skin is critical for our understanding of rickettsiosis.

**Methodology/Principal Findings:**

Here, we report the first genome-wide analysis of *R. conorii* gene expression from infected human skin biopsies. Our data showed that *R. conorii* exhibited a striking transcript signature that is remarkably conserved across patients, regardless of genotype. The expression profiles obtained using custom Agilent microarrays were validated by quantitative RT-PCR. Within eschars, the amount of detected *R. conorii* transcripts was of 55%, this value being of 74% for bacteria grown in Vero cells. In such infected host tissues, approximately 15% (*n* = 211) of the total predicted *R. conorii* ORFs appeared differentially expressed compared to bacteria grown in standard laboratory conditions. These genes are mostly down-regulated and encode proteins essential for bacterial replication. Some of the strategies displayed by rickettsiae to overcome the host defense barriers, thus avoiding killing, were also pointed out. The observed up-regulation of rickettsial genes associated with DNA repair is likely to correspond to a DNA-damaging agent enriched environment generated by the host cells to eradicate the pathogens. Survival of *R. conorii* within eschars also involves adaptation to osmotic stress, changes in cell surface proteins and up-regulation of some virulence factors. Interestingly, in contrast to down-regulated transcripts, we noticed that up-regulated ones rather exhibit a small nucleotide size, most of them being exclusive for the spotted fever group rickettsiae.

**Conclusion/Significance:**

Because eschar is a site for rickettsial introduction, the pattern of rickettsial gene expression observed here may define how rickettsiae counteract the host defense.

## Introduction

*Rickettsia conorii* is a Gram-negative bacterium responsible for the Mediterranean spotted fever (MSF), a disease transmitted to humans by the brown dog tick *Rhipicephalus sanguineus* [1]. Inoculation of rickettsiae to human beings leads to vasculitis and lesions at the site of tick bite [2]. The cutaneous necrosis that results from severe injury to many small vessels and otherwise called the “tâche noire” is the hallmark of many spotted fever group rickettsioses [3]. The histological examination of eschars collected from patients suffering from boutonneuse fever indicated that the alterations were mainly located in the dermis and subcutaneous tissues and evidenced the presence of rickettsiae in blood vessels [3]. Thus, indirect immunofluorescent detection of *R. conorii* on cryostat sections of skin biopsy specimens from patients was found to improve the early diagnosis of severe and atypical forms of MSF [4], [5].

Inoculation eschars correspond to the portal of entry of the infectious agent into the host and the first site of challenge between the infected human being and the bacterium. Within the first 24 hours after the tick attachment, the rickettsiae are already blood-borne and the observed rickettsiae in the tick feeding site and in particular within the eschar are left over rickettsiae undergoing clearance [2]. In this respect, the “tâche noire” was depicted as being an excellent, accessible model for the study of the human-*Rickettsia* interaction [6]. The intralesional expression of local mediators of inflammation and of immune response that could contribute both to anti-rickettsial immunity and the pathogenesis of the MSF, has recently been depicted in infected human tissues [7]. Analysis of the complementary picture, namely the survival strategies used by *R. conorii* within the inoculation site should provide a better understanding of rickettsial pathogenesis. While reports of global gene expression profiling in human tissue or non-invasive patient samples suffering from bacterial diseases are limited [8], [9], we explored the RNA profiles of *R. conorii* from eschars collected on MSF patients. This study was made possible by applying a strategy combining removal of eukaryotic contaminants with subsequent random amplification of prokaryotic cDNA [10] that was found convenient for microarray-based transcriptome analysis of obligate intracellular rickettsiae [11]. To identify genes differentially regulated within eschars, rickettsial microarrays hybridized with cDNA obtained from *R. conorii* grown in Vero cells monolayers at 32°C were used as control. The results obtained offer new insights into *R. conorii* survival within the eschar site.

## Results

### The transcriptome profile of *R. conorii* is highly conserved among different eschars

**Figure 1:**
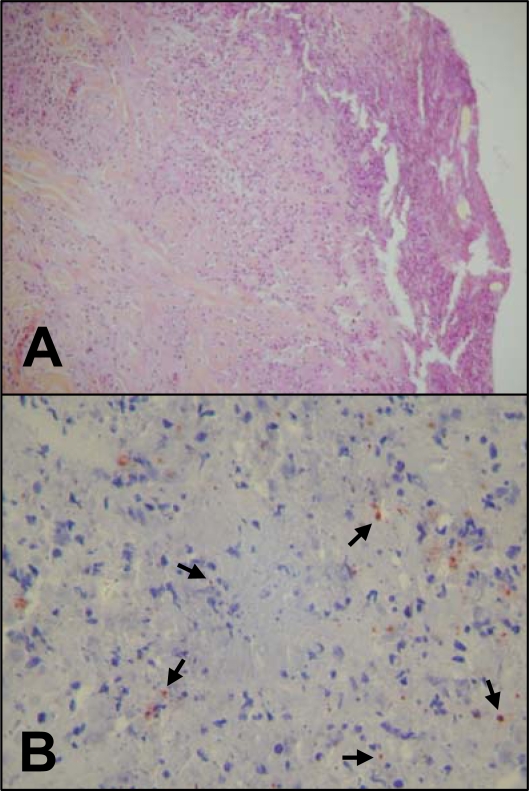
Inoculation eschar from a patient with MSF. (A) Histologic examination of inoculation eschar from a patient with MSF showing numerous dermal inflammatory infiltrates mainly composed of polymorphonuclear leukocytes with large necrotic areas and coagulative necrosis of the epidermis (hematoxylin-eosin-saffron, original magnification ×100). (B) Immunohistochemical detection of *R. conorii* in the inoculation eschar. Anti-*R. conorii* antibodies were detected using biotinylated secondary antibody, followed by avidin-peroxidase color development. The bacteria thus stained in reddish/brown, and indicated by the arrows, appear located between the necrotic inflammatory cells present in the dermis (original magnification ×400).

**Figure 2:**
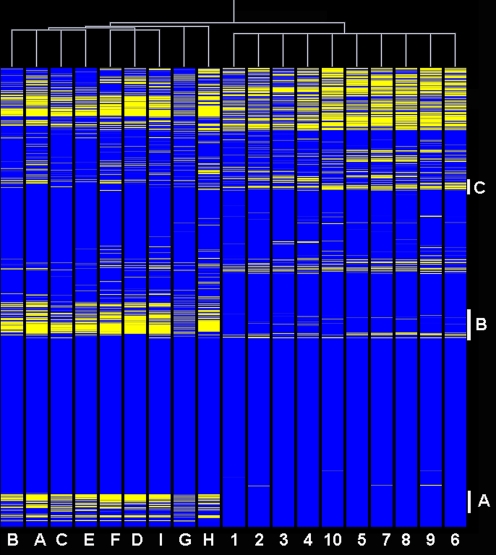
Two-way clustering analysis of *R. conorii* transcriptomic patterns. The figure shows a clear separation of two clusters containing 8 eschars recovered from different infectious episodes and 10 slides corresponding to bacteria grown in Vero cells and used as control, respectively. Each probe set is represented by a single row of colored boxes and each sample (eschar or control) correspond to a single column. The blue areas correspond to genes showing high or medium expression whereas yellow bars indicates genes poorly or not expressed. The dendrogram (white lines) on the top of the figure represents the similarity matrix of probe sets. Among clusters of genes allowing discriminating between the 2 tested conditions, A contains 70 genes involved mainly in different metabolic functions (transporters, DNA repair enzyme *mutL*, metabolic enzymes and numerous ribosomal proteins), cluster B contains an important number of genes involved in energy production (ATP synthesis) as well as genes involved in stress-response (*uvrA* and C and *htpG*), cell division and some virulence factors (*virB4* and *virB10*) and antibiotic resistance determinants. Cluster C contains several hem factors.

The bacterial RNA samples purified from 8 eschars derived from 7 individual patients (Table 1) were subjected to whole-genome-wide transcript expression profile analysis and compared to the transcriptome of *R. conorii* grown *in vitro* and used as reference. As illustrated by Figure 1, human skin biopsies of MSF patients are histologically dominated by severe cutaneous necrosis with coagulative necrosis of the epidermis. Such specimens contain *R. conorii*, the bacteria being mostly found in necrotic areas associated with inflammatory cells. Given the scarce amount of available material, only one sample was hybridized twice. Hierarchical clustering of the signal intensities of the individual transcripts in both groups evidenced a high similarity of transcript expression patterns among eschars or infected cell monolayers, respectively. Measurements derived from similarity matrix indicated that largest distance among Vero cells profiles was 0.056 whereas a distance of 0.188 was found for the different eschars (Fig. 2). To accurately assess variations in gene expression within the group 〈〈eschar〉〉, the phylogenic analysis of the different clinical isolates was achieved with the multispacer typing (MST) based on the sequences of 3 variable intergenic spacers, namely *dksA-xerC*, *mppA-purC*, *rpmE-tRNA(fMet)* [12]. By combining the genotypes obtained from these three intergenic spacers, only 3 MST genotypes were obtained (Table 1). It is interesting to note that the two *R. conorii* isolates for which the transcriptome profiles appeared divergent from the main cluster (samples G and H) exhibited distinct MST genotypes.

**Table 1:** Clinical characteristics of patients included in this study.

sample	age	sex	number of eschars	year of eschar sampling	severe form	geographical site of bite	MST genotype	strain isolated
E	62	F	1	2005	No	France (13)	Nd	no
G	42	F	1	2005	No	Marocco	C	no
C	72	F	1	2004	Yes	France (84)	A	yes
I	67	M	1	2005	Yes	France (13)	A	no
F,D	61	F	2	2004	Yes	France (13)	A	yes
A,B	45	M	1	2006	No	France (13)	A	yes
H	30	M	1	2005	No	Algeria	B	yes

(nd) not determined.

**Figure 3:**
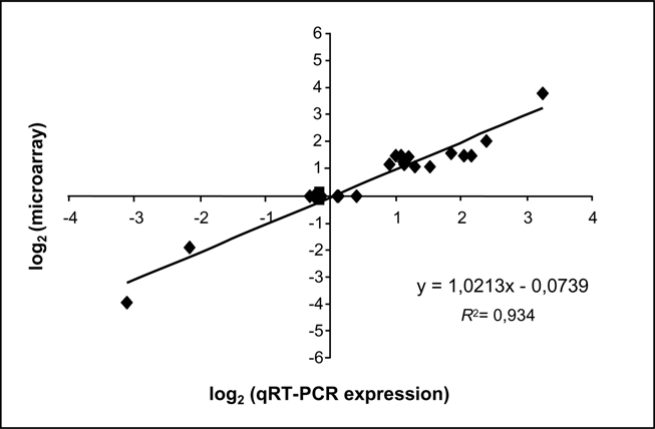
Validation of microarray-based expression profiles by qRT-PCR. The relative transcriptional levels for 16 genes were determined by real-time qRT-PCR using cDNA as template. Following normalization of data based on values measured with non regulated transcripts, the real-time qPCR log_2_ values were plotted against the microarray log_2_ values. The correlation coefficient (R^2^) for comparison of the two datasets is of 0.934.

The microarray results were confirmed by qRT-PCR for a subset of 16 targets. When comparing both methods, a high correlation coefficient (R^2^ = 0.934) was observed (Fig. 3).

### General overview

When compared with bacteria grown in Vero cell monolayers, the *R. conorii* gene transcripts in eschars were mainly found to be down-regulated. Of the 5,098 probes represented on our microarrays, 211 transcripts representing 15.4% (211/1374) of the *R. conorii* ORFs were differentially expressed (*P*<0.05). Of these, 180 genes were down-regulated two-fold or greater *in vivo* (supplementary Table S2) while only 31 genes exhibited an increased expression (Table 2). In the *R. conorii* genome, the size of the 1,374 annotated ORFs is ranging from 120 to 6,063 nucleotides (nt) with a mean value of 804 nt [13]. We noticed that 87% (27/31) of up-regulated genes within eschars have a size ranging from 153 nt to 723 nt, being thus significantly smaller than the median value. We also observed that 21 of these genes, i.e. 67.7% are lacking or highly degraded in the typhus group (TG) rickettsiae. In contrast, the mean size of down-regulated genes was of 1,323 nt and 75% of them belong to the core gene of rickettsiae [14].

**Table 2:** Thirty one genes of *R. conorii* up-reguled within eschars.

ORFs	Genes	Description	Fold-Ch	Size (nt)
RC0550		RecB family exonuclease	13,685	2,517
RC0828**		**Unknown**	**6,763**	**183**
RC0500		**ABC transporter ATP-binding protein**	**4,019**	**723**
RC1370*		**Prophage antirepressor (SPLIT GENE)**	**3,790**	**207**
RC0769**		**Unknown**	**3,661**	**156**
RC1137	*phbC*	**Poly-beta-hydroxybutyrate polymerase (SPLIT GENE)**	**3,628**	**264**
RC1298**		**Lysozyme (FRAGMENT)**	**3,405**	**204**
RC0350**		**Unknown**	**3,225**	**240**
RC1204	*smpA*	**tmRNA-binding protein**	**3,068**	**474**
RC0299*	*mdlB*	**ABC-type multidrug transport system, ATPase and permease components (SPLIT GENE)**	**2,986**	**273**
RC1299**		**Unknown**	**2,972**	**165**
RC0921**		**Unknown**	**2,825**	**153**
RC1349**		**Unknown**	**2,814**	**201**
RC0637	*trxB2*	**Thioredoxin reductase [EC:1.6.4.5]**	**2,785**	**1,023**
RC0267	*grxC1*	**Glutaredoxin**	**2,780**	**309**
RC0461*		**Glycosyltransferase [EC:2.4.1.-], two domains**	**2,708**	**1,815**
RC1050**		**Transposase (FRAGMENT)**	**2,655**	**171**
RC1125*		**Superfamily I DNA and RNA helicases (SPLIT GENE)**	**2,617**	**192**
RC0378	***nuoL3***	**NADH dehydrogenase I chain L [EC:1.6.5.3]**	**2,507**	**1,518**
RC1251		**Unknown**	**2,390**	**504**
RC0221**	***cmcI***	**Cephalosporin hydroxylase (FRAGMENT)**	**2,342**	**321**
RC0914		**Tetratricopeptide repeat-containing protein**	**2,271**	**264**
RC0957*		**Tetratricopeptide repeat-containing protein (FRAGMENT)**	**2,263**	**228**
RC1155*		**Unknown (SPLIT GENE)**	**2,256**	**348**
RC0209*		**Unknown**	**2,225**	**282**
RC0795**		**NACHT family NTPase (FRAGMENT)**	**2,146**	**213**
RC0890*	*pro*P9	Proline/betaine transporter (FRAGMENT)	2,102	515
RC0269	*atm1*	Multidrug resistance protein Atm1 (SPLIT GENE)	2,085	201
RC0060**		**Unknown**	**2,067**	**492**
RC1144**		**Unknown**	**2,066**	**231**
RC1192**		**Putative AAA+ superfamily ATPase (SPLIT GENE)**	**2,061**	**183**

Genes absent (**) or degraded (*) in the TG.

### Evidence for rickettsial growth arrest within eschars

**Figure 4:**
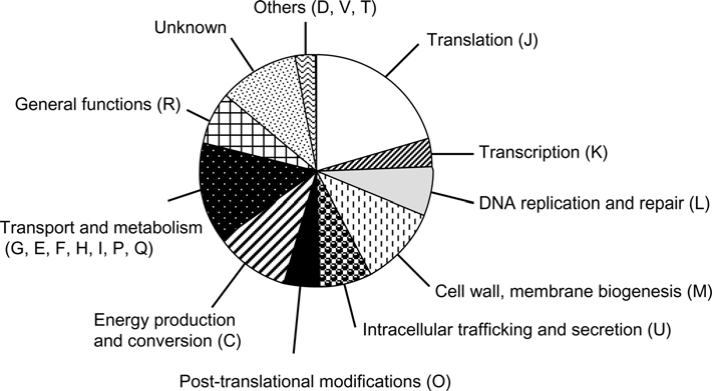
Distribution of *R. conorii* ORFs down-regulated during infection. Pie chart showing the percentages of transcripts down-regulated in eschars and classified according to their respective functional categories. COG category legend is as follow: Cell cycle control, mitosis and meiosis (D); Defense mechanisms (V); Signal transduction mechanisms (T); Transport and metabolism of carbohydrates (G), amino acids (E), nucleotides (F), coenzymes (H), lipids (I), inorganic ions (P), and of secondary metabolites (Q).

The ORFs of *R. conorii* whose expression was significantly altered were classified into functional categories according to the Cluster of Orthologous Gene (COG) classification, as defined by the COGs database [15]. As illustrated Figure 4, the most down-regulated COGs were translation (J), cell wall and membrane biogenesis (M), intracellular trafficking and secretion (U) as well as energy production and conversion (C). The reduced expression of 24 genes encoding ribosomal proteins indicates that, within eschars, rickettsiae are reducing their translational capacity (supplementary Table S2). Eighteen of these genes (from RC0981 to RC1007) are grouped together on the rickettsial chromosome and are organized as a ribosomal protein gene cluster. Consistent with a bacterial growth arrest, we also noticed a dramatic decrease in the expression of cell wall components including several Sca family proteins (Sca0, Sca1, Sca4, Sca5 and Sca10). Following 23SrRNA (105.5-fold decrease), the most down-regulated genes are those encoding the rickettsial outer membrane protein (rOmpB otherwise called Sca5: 98.5-fold decrease) and the rickettsial adhesin Adr2 (79.7-fold decrease). While down-regulated in a lower extend (fold-change mean of 5.4), many of the variable genes from the U category, including VirB, SecE, SecF and SecY subunits, encode proteins associated with the cell membrane. We also noticed the down-regulation of genes involved in energy production that should also contribute to bacterial replication arrest. Finally, 18% of down-regulated genes have no clearly defined function (S and R) or are not assigned to any functional categories.

### *R. conorii* response to the host attack

Among the 31 rickettsial genes up-regulated in eschars, several are plausibly involved to escape host response. They include genes coding for proteins involved in DNA repair and modification as RC0550 (RecB exonuclease), RC1204 (tmRNA-binding protein), RC1050 (transposase) and RC1125 (helicase). Bacteria also face to oxidative stress, as indicated by the increase expression of *phbC* (poly-beta-hydroxybutyrate polymerase), *trxB2* (thioredoxin) and *grxC1* (glutaredoxin). An enhanced tolerance to osmotic stress could be provided through the increased expression of *proP9* (proline-betaine transporter) and *nuoL3* (NADH dehydrogenase chain I). Adaptation of *R. conorii* within eschars promoted changes in cell-wall related proteins as glycosyltransferase and cephalosporin hydroxylase (*cmcl*) and was accompanied with the up-regulation of several ATP binding cassette (ABC) transporters (RC0500, *mdlB*, *atm1*) and of two proteins that belong to the KAP NTPases, namely the NACHT NTPase and the putative AAA+ family ATPase, a new family characterized by the presence of transmembrane segments inserted into the P-loop NTPase domain [16]. We also noticed the over-expression of two genes encoding tetratricopeptide repeat (TPR)-containing proteins, suggesting the importance of this motif for protein-protein interactions between *R. conorii* and its host during infection, as previously evoked [17]. Apart from these, we also observed an increased expression of RC1370 and RC1298 genes that encode a prophage antirepressor and lysozyme, respectively. Finally, 11 out of the 31 up-regulated genes encode proteins with unknown function.

## Discussion

Infection with *R. conorii* usually occurs following infected tick bite [2] and the first step of this host-pathogen interaction takes place at the inoculation site where anti-rickettsial immunity is enhanced [7]. The presence of an eschar in rickettsial disease is generally linked with a milder disease as more severe rickettsiosis, namely the Rocky Mountain Spotted Fever (RMSF) and epidemic typhus do not exhibit inoculation eschars [1], [2], [18]. This suggests that eschar corresponds to the front line of human host defense against rickettsia diffusion, a point consistent with the fact that the highest bacteremia were detected in patients suffering from RMSF and typhus [19]. Accordingly, the factors involved in the bacterial survival strategy can be considered as crucial actors of *R. conorii* pathogenesis. We thus examined the expression patterns of *R. conorii* in such environment, using an approach successfully applied for the analysis of transcriptional profile of bacteria within infected epithelial monolayers [11].

The human skin biopsies being rare and precious, the optimal conditions for total RNA extraction were first assayed on eschars experimentally induced through the intradermal injection of *R. conorii* in rabbits (not shown). Starting material used in this study was collected at different time points after the tick bite and stored at −80°C for variable periods of time (up to 3 years) before processing. We predicted that this factor would interfere with the identification of differences in gene expression because of biological variations. In addition, the pattern of gene expression may also vary depending on the genetic background of infecting strains. Indeed, in microarray-based experiments, a lower hybridization can result either from a reduced amount of transcripts or from divergence in the sequence of the gene [20]. Thus, insertion or deletion events in the genome of clinical strains tested could affect the apparent transcript abundance measured by using a microarray designed from the genome sequence of the Malish strain (seven) of *R. conorii* [13]. Here, three *R. conorii* strains corresponding to cases issued from three different geographical sites were identified. However, analysis of rickettsial transcript expression patterns from the 8 human eschars included in this study yielded reproducible results and only minor variations were observed between biopsy specimens. Finally, results obtained by qRT-PCR indicated that reliable microarray hybridizations can be achieved with rickettsial RNA extracted from multiple and independently obtained eschars.

This work showed that within eschars, the *R. conorii* transcripts were mainly down-regulated compared to bacteria internalized in Vero cells. The most significantly repressed genes are those of the translation machinery. This observation is consistent with previous analysis of the transcriptional changes displayed by 19 different bacterial pathogens upon eukaryotic cell infection [8]. Within these conditions, a general decline of genes involved in general metabolism associated with bacterial growth (translation, transcription, cell wall biogenesis, energy production, transport of carbohydrates, amino acids and nucleotides) was observed. Such a global expression decrease resembles that depicted for *Bacillus subtilis* [21], *Escherichia coli* [22], *Corynebacterium glutamicum* [23], and *Staphylococcus aureus* [24] after inducing the stringent response [25]. The recent analysis of inflammatory and immune mediators present in skin-biopsy samples of patients suffering from MSF evidenced the production of some enzymes including inducible nitric oxide synthase and indoleamine-2,3-dioxygenase, that should contribute to the bacterial growth arrest [7]. However, we observed that in some cases, the immune and inflammatory host response was therefore not strong enough to eradicate all infecting *R. conorii* as the bacterium was cultivable from 5 (62.5%) of the eschar biopsy specimens.

Analysis of obtained data highlighted some of the mechanisms displayed by the rickettsiae to counter the damage and survive within the host cells. Genes encoding proteins involved in genome repair and allowing bacteria to cope with DNA-damaging agents are probably critical in this situation and were up-regulated. DNA repair is a fundamental process used by pathogenic bacteria as one of the defense mechanisms that allow them to survive in their hosts [26]. As described for *Helicobacter pylori* [27], the DNA lesions could result from a cellular oxidative stress, a point consistent with the increased rickettsial defenses against reactive oxygen species. Noteworthy is that production of bactericidal reactive oxygen species is one of the key methods by which mammalian infected cells efficiently kill bacteria [28]. Within eschars, *R. conorii* also deal with osmotic stress as indicated by the up-regulation of proline-betaine transporter and of NADH dehydrogenase I. Proline and betaine are two osmoprotectants accumulated through enhanced uptake rather than synthesis by Gram negative bacteria to overcome the inhibitory effects of hyperosmolarity [29]. While the *nuo* genes of rickettsiae are more closely related to mitochondria than to any other studied microbe [30], enhancement of NADH dehydrogenase expression could correspond to another strategy of osmoadaptation evolved to achieve salt tolerance. *R. conorii* also displayed another hallmark feature of pathogens interacting with host cells, namely the phenotypic changes in the composition of several membrane proteins [8]. It is well established that antigenic variation is an important mechanism that allows pathogens to escape for immunity. In this respect, we noticed an important down-regulation of genes encoding for the Sca family proteins among which Sca0 (rOmpA) and Sca5 (rOmpB) which are two major rickettsial antigenic determinants [31]. Antigenic variation could also be related to the post-translational modifications of proteins. These changes could be afforded by Cmcl, a protein recently classified as a methyltransferase based on structural evidences [32], or by a glycosyltransferase. Because most glycoproteins were associated with virulence factors in bacterial pathogens [33], these events could contribute to differences in both virulence and antigenicity of *R. conorii in vivo*.

**Figure 5:**
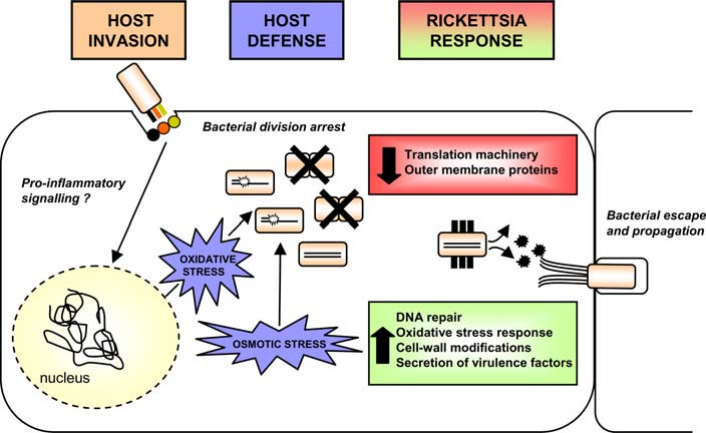
Schematic representation of the interaction between *R. conorii* and its host cell at the site of inoculation. Intracellular uptake of *R. conorii* by induced phagocytosis [46] may activate signalling pathways in the host cells which in turn display several mechanisms to eliminate invading bacteria among which oxidative and osmotic stress. As shown by the work presented in this paper, these events induce a strong down-regulation of *R. conorii* transcripts, mainly of those involved in bacterial replication and classified within translation and cell wall membrane COGs. These pathogenic bacteria therefore evade the host defense through the up-regulation of several factors counteracting DNA damages and through variations of dominant surface antigens allowing to avoid host recognition. The secretion of virulence determinants is also likely to favour survival and colonization of the host.

Clearly, besides evasion of host defense, rickettsiae also exhibited virulence determinants *in vivo*. Thus, the export of virulence proteins in the host cell cytoplasm could be achieved through the increased expression of ABC transporters that function as type I secretion system in Gram negative bacteria [34]. The way in which rickettsiae use the KAP NTPases in the intracellular host cell environments has not yet been investigated but several members of this family, namely the AAA+ ATPases were described to promote virulence of other bacterial pathogens [35]. A role for the α-superhelical structure domain of KAP NTPases has been evoked in protein-protein interactions [16]. Interestingly, such interactions can also be mediated by the TPR [36], another structural motif present within two *R. conorii* proteins up-regulated *in vivo*. Here again, the functional role of these ubiquitous domains was not deciphered. However, TPR repeat regions have been implicated in the ability of *L. pneumophila* to efficiently establish infection and/or to manipulate host cell trafficking event [37]. The whole picture of this host-pathogen interaction within the first step of infection is summarized Figure 5.

A more general view of obtained results showed that, to ensure microbial fitness and survival in the lethal host environment, *R. conorii* mainly promoted the transcription of small size genes. While a high proportion of them codes for proteins with unknown functions, those with functional attributes including *phbC*, *mdlB* and *proP9* have already been associated with pathogenicity in other bacteria. Another interesting feature is that up-regulated genes are mostly exclusive for SFG rickettsiae, a finding consistent with the fact that the inoculation site corresponds to environmental conditions not encountered by TG bacteria that are transmitted to human by exposure to feces of infected lice or fleas [1].

Recent studies showed that an understanding of the basic mechanisms of adaptation of rickettsiae under various environments, including nutrient deprived medium [11] and temperature changes [38] can be gained by determining their global transcriptome profile by microarrays. As shown here, this approach also offers the opportunity to characterize strategies displayed by the bacteria for *in vivo* survival. A new picture of *Rickettsia* pathogenicity, still poorly known, has thus emerged. A better knowledge of such an host-pathogen interaction would offer the opportunity to identify future therapeutic targets usefull for the prevention or the treatment of rickettsiosis.

## Materials and Methods

### Human subjects and sample collection

From June 2004 through August 2006, cutaneous biopsy specimens were obtained from seven patients in Marseille with eschar-associated illnesses, and who were suspected of suffering from MSF on the basis of initial clinical findings or laboratory analysis. These biopsy specimens, excised by using a scalpel, were dissected in two pieces. For RNA extraction purpose, the tissues were collected in sterile Eppendorf tubes containing RNA*later* Stabilization Reagent (Qiagen, Courtaboeuf, France) refrigerated at 4°C overnight before storage at −80°C. The second half of specimens was processed for bacterial culture and PCR-based molecular diagnosis, histopathology as well as immunohistochemical staining after fixation in ethanol. Selected clinical variables of the individual patients are shown in Table 1. These experiments were carried out with both the approval from the local ethics committee (IFR 48 ethics committee) and the written consent of informed patients.

### Histologic analysis and immunohistochemical detection of *R. conorii*

Formalin-fixed, paraffin-embedded skin biopsy specimens of the inoculation eschars were cut (3 µm thickness) and stained with hematoxylin-eosin-saffron by routine staining methods. Serial sections of each tissue specimen were also obtained for immunohistochemical investigations. The immunohistochemical analysis was performed by the indirect immunoperoxidase method has described elsewhere [39] and using a polyclonal rabbit antibody against *R. conorii* as primary antibody. Briefly, after deparaffinization, each tissue section was incubated with the polyclonal anti-*R. conorii* antibody (1∶2000) and immunodetection was performed with biotinylated immunoglobulins, followed by peroxidase-labeled streptavidin (HistoStain plus kit, Zymed, Montrouge, France) with amino-ethyl-carbazole as substrate. The slides were counterstained with Mayer hematoxylin for 10 min. Images were acquired with a Zeiss Axioskop microscope coupled with a Nikon Coolpix 4500 digital camera.

### Multispacer genotyping (MST) of *R. conorii* clinical isolates

Total genomic DNA was extracted from skin-biopsy specimens using the QIAamp Tissue kit (Qiagen), as described by the manufacturer. Amplification of the three highly variable intergenic spacers, *dksA-xerC*, *mppA-purC*, and *rpmE-tRNA^fMet^* was achieved using previously described primers [12] and HotStarTaq DNA polymerase (Qiagen). All primers were obtained from Eurogentec (Seraing, Belgium). PCR was carried out in a PTC-200 automated thermal cycler (MJ Research, Waltham, Mass.) under the following conditions: an initial 15 min-step at 95°C was followed by 39 cycles of denaturation for 30 s at 94°C, annealing for 30 s at 54°C, and extension for 1 min at 72°C. Final amplification was completed by holding the reaction mixture for 5 min at 72°C to allow complete extension of the PCR products. Following purification (MultiScreen PCR filter plate, Millipore, Saint-Quentin en Yvelines, France), amplicons were sequenced in both directions using the BigDye 1.1 chemistry (Applied Biosystems) on an ABI 3130XL automated sequencer (Applied Biosystems) as described by the manufacturer. To avoid contamination, no positive control was used. Sterile water was used as a negative control in each PCR assay. Sequences from each DNA sample were checked twice in both directions to ensure the reliability of the MST method, then assembled and edited with the Sequencher 4.7 program (GeneCode, Ann Arbor, Mich.). For the phylogenetic analysis, the sequences of three spacers were concatenated. Multiple alignments of the concatenated spacer sequences were carried out using the CLUSTALW software [40]. Phylogenetic relationships were obtained using the neighbor-joining and maximum parsimony methods within the MEGA 4.1 software [41].

### Microarray design

ArrayDesigner™ (Premier Biosoft) was used to generate an initial set of probes covering the whole genome of *R. conorii* strain Malish 7 (NC_003103), showing specific physico-chemical properties: (i) 71±12° C target Tm, (ii) 40–60 bp probe length, (iii) <−5.0 kcal/mol for hairpins, (iv) <−8.0 kcal/mol for self-dimers, and (v) dinucleotide repeats shorter than 5 bp. Candidate probes were tested for specificity against the aforementioned *R. conorii* genome using Olicheck [42]. To avoid cross-hybridization with host cell nucleic acids, the resulting set of probes was subjected to Blast analysis against the *Homo sapiens* and *Pan troglodytes* (by default of the African green monkey genome, Vero cells). Probes with >18 consecutive nt matches were excluded. Preceding steps yielded a final oligonucleotide set of 5,098 probes resulting to a final coverage of 97% for ORFs and 63% for inter-ORFs (considering fragment with length >149 bp which is the median size of inter-ORFs). To minimize steric hindrance, all probes <60 nt in length were poly(T)-tailed to reach an overall length of 60 nt, following Agilent's recommendations. Microarrays (2×11K format) were manufactured by *in situ* synthesis SurePrint technology (Agilent Technologies, Palo Alto, CA). All specific oligonucleotides as well as Agilent's control spots were printed in duplicate.

### RNA isolation and purification from eschars

Total RNA extraction and purification from the eschar biopsies was carried out using the RNeasy kit (Qiagen) with some modifications. Briefly, the tissue samples removed from RNA*later* were rapidly decontaminated with iodated alcohol. After 2 washings in RNase-free water, 20 mg of tissue excised in small pieces were homogenized in RLT solution with tungsten beads and using the Mixer Mill MM300 (Qiagen). The resulting homogenate was then incubated at 55°C for 10 min with proteinase K (200 µg/ml) and centrifuged for 3 min at 10,000×*g*. Supernatant containing the total RNA fraction was supplemented with ethanol and purified onto RNeasy columns according to the manufacturer's instructions. The amount and quality of obtained RNA were determined with the microfluidic-based platform (Agilent 2100 Bioanalyzer) and using the RNA 6000 Nano Labchip kit (Agilent). In the electropherograms obtained with total RNA, the prokaryotic fraction was not always detected because its low abundance and the profiles of eukaryotic peaks were used as indicators for the integrity of both RNA populations. Estimated amount of total RNA extracted from 20 mg eschar was generally around 10 µg, including 90% of eukaryotic RNAs. These contaminants were removed using the MICROBEnrich procedure (Ambion, Applied Biosystems, Courtaboeuf, France) and prokaryotic cDNA was synthesized as described [10] using the M-MLV Reverse Transcriptase (Invitrogen, Cergy-Pontoise, France).

### RNA isolation and purification from infected Vero cells

*R. conorii* strain Malish 7 (ATCC, VR613) grown in Vero cells for 48 h at 32°C were lysed in TE buffer (10 mM Tris/HCl, 1 mM EDTA pH 7.0) supplemented with lysozyme (10 mg/ml) for 10 minutes at room temperature. Total RNA was then extracted and purified using RNeasy-Midi columns (Qiagen), as previously described [11]. Two batches bacteria grown separately (biological replicates) were collected for this study. We then applied the same procedure as for eschar samples for purification, amplification, labelling and hybridization of *R. conorii* cDNA.

### Expression microarrays and analysis

Twenty nanograms of cDNA were amplified using the GenomiPhi DNA amplification kit (Amersham Biosciences, Uppsala, Sweden) and labeled with Cy3-dCTP or Cy5-dCTP dyes (Amersham Biosciences) using the BioPrime DNA labeling System (Invitrogen, Cergy-Pontoise, France). Following purification with GFX columns (Amersham Biosciences), the levels of Cy3-dCTP and Cy5-dCTP incorporation were quantified by absorbance measurement at 550 nm and 650 nm, respectively. Hybridizations were performed for 17 h at 60°C in dedicated micro-chambers with 75 pmol of both control or eschar samples. Stringent washings were then performed according to manufacturer's instructions. Slides dried by 30 sec washing with acetonitrile were scanned using 100% Photon Multiplier Tube power for both wavelengths using Agilent scanner (Agilent Technologies, CA, USA) and extracted using Feature Extraction™ software (version 8, Agilent). Local background subtracted signals were corrected for unequal dye incorporation or unequal load of the labeled product and normalized using GeneSpring (Agilent). The algorithm consisted of a rank consistency filter and a curve fit using the default LOWESS (locally weighted linear regression) method. Data consisting of 10 independent control conditions hybridized against 10 independent patient eschar experiments were expressed as Log10 ratios and analyzed using GeneSpring 7.3 (Agilent). Statistical significance of differentially expressed genes was identified by variance analysis (ANOVA) [43], [44] performed using GeneSpring, including the Benjamini and Hochberg false discovery rate correction (5%). Expression microarrays (normalized data) were clustered by a hierarchical clustering algorithm by using an average linkage method in GeneSpring. The expression values for a gene across all samples were standardized to have mean of 0 and standard deviation of 1 by linear transformation. To determine the amount of detectable genes, the expression values were averaged for transcripts mapped by 2 or more probes. A cut-off value defined as 2×standard deviation obtained for background intensities was then applied [45].

### Microarray data accession numbers

The data have been deposited in NCBI Gene Expression Omnibus (GEO; http://www.ncbi.nlm.nih.gov/geo/). The GEO accession numbers are GPL7040 for the platform of the microarray and GSE12130 for the experimental data set.

### Real-time quantitative PCR

Validation of microarray data was achieved using cDNA synthesized from eschar specimens A/B, C and E and from *R. conorii* grown in Vero cells used as reference. Real-time quantitative RT-PCR (qRT-PCR) was performed on the Smart-Cycler system (Cepheid) together with the QuantiTec Probe PCR kit (Qiagen) or SybrGreen DNA Fast Start kit (Roche Diagnostics, Basel, Switzerland), as indicated. Gene-specific primers are listed in the Supplementary Table S1. The values obtained for the 3 non-differentially expressed genes (*trxB1*, *glyQ* and *dnaK*) were used to normalize all data. The fold change (FC) in expression of the target genes relative to the 3 unregulated genes was determined as follows: FC = 2^−ΔΔCt^ where ΔΔCt = (Mean-Ct_target_−Mean-Ct_control_)_eschars_−(Mean-Ct_target_−Mean-Ct_control_)_reference_. Ct values were defined as the cycle numbers at which the fluorescence signals were detected.

## Supporting Information

Table S1 List of primers and conditions for qRT-PCR assays
(0.04 MB XLS)

Table S2 R. conorii genes down-regulated within eschars
(0.08 MB XLS)
